# Supplementary material for: Combining p53 mRNA nanotherapy with immune checkpoint blockade reprograms the immune microenvironment for effective cancer therapy
Source: Nat Commun. 2022 Feb 9;13:758. doi: 10.1038/s41467-022-28279-8 (PMC8828745; doi:10.1038/s41467-022-28279-8)
Supplement: Supplementary file 1 — Supplementary Information [file 41467_2022_28279_MOESM1_ESM.pdf]

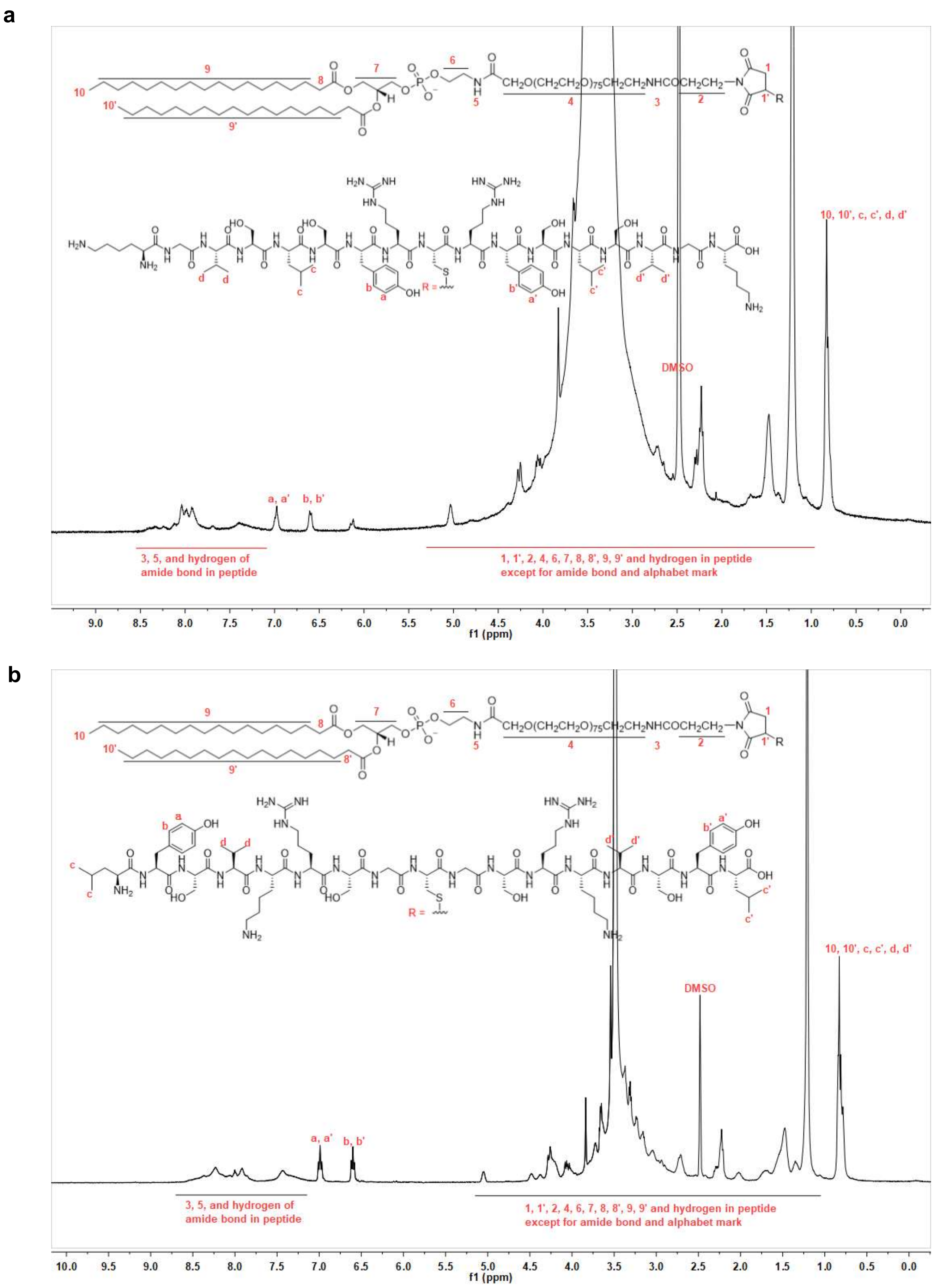

Supplementary Figure 1. The chemical structure and ^{1}H-NMR spectra of **(a)** DSPE-PEG-CTCE and **(b)** DSPE-PEG-SCP.

a

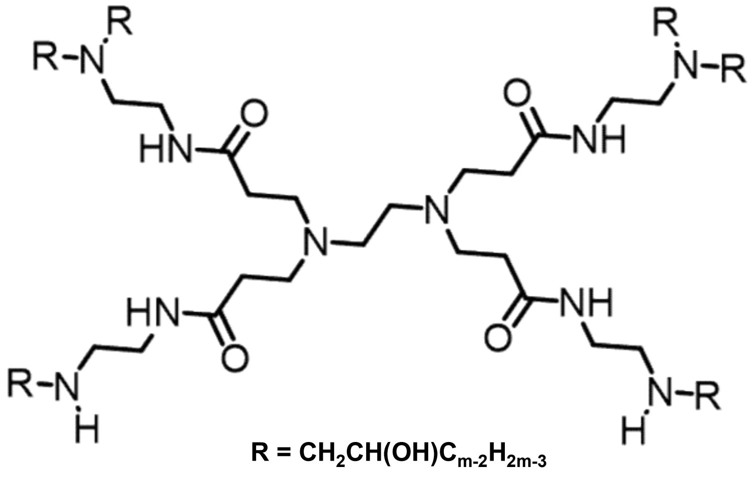

b

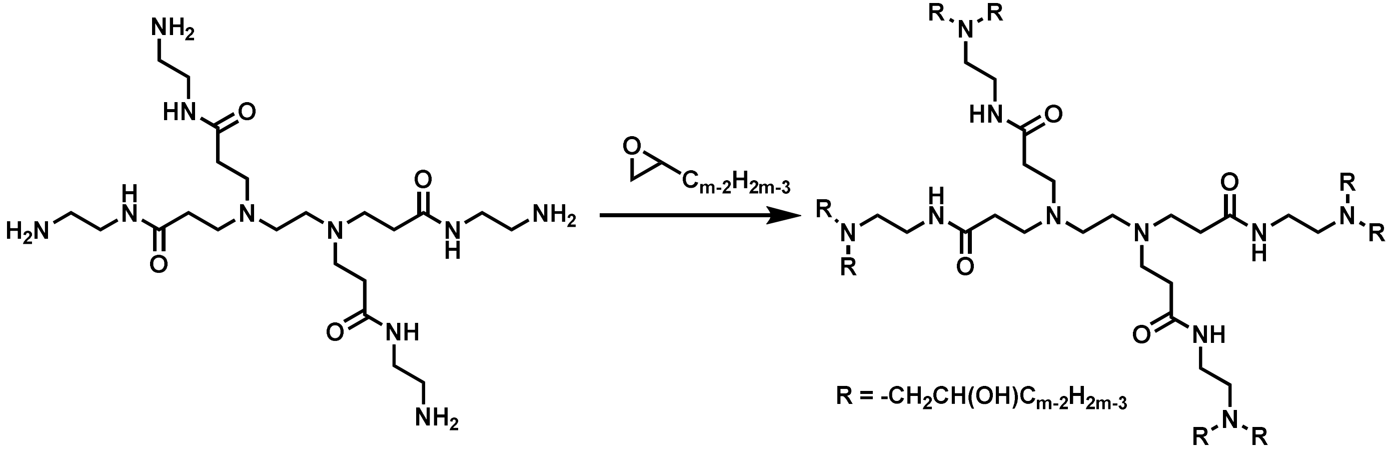

Supplementary Figure 2. (a) The chemical structure and **(b)** chemical synthetic route of different cationic lipids.

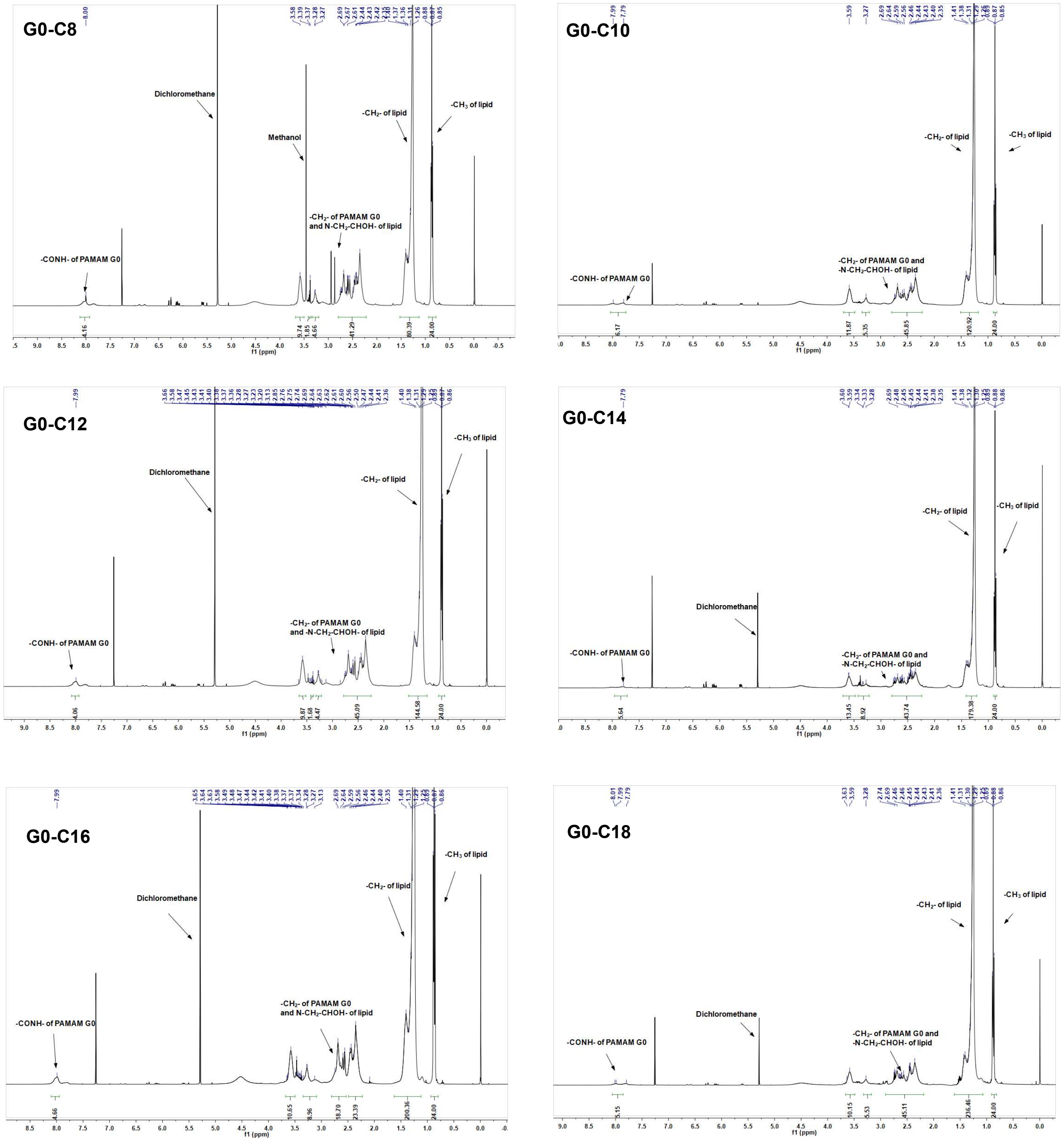

Supplementary Figure 3. ^{1}H-NMR spectra of different cationic lipids.

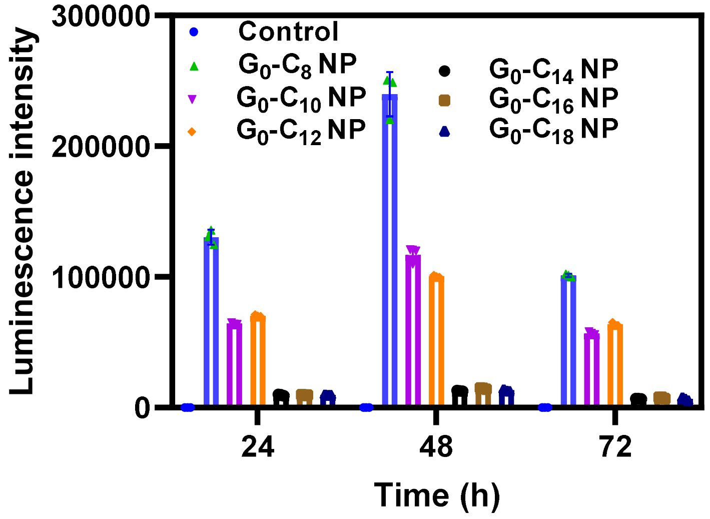

Supplementary Figure 4. Effect of different cationic lipid-like materials G0-Cm on the transfection efficacy of Luciferase-mRNA NPs (mRNA concentration: 0.5 μg/mL). All data are presented as mean ± S.D. This experiment was repeated thrice independently with similar results.

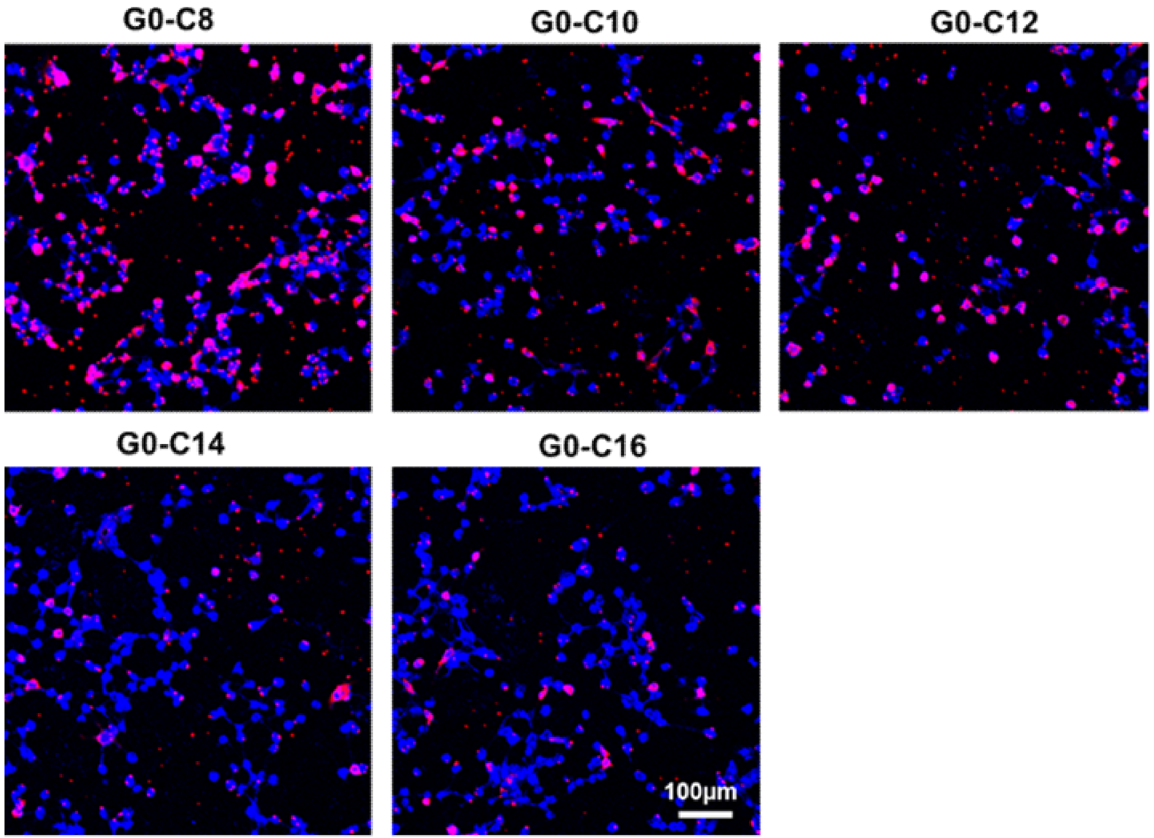

Supplementary Figure 5. Effect of different cationic lipid-like materials G0-Cm on the cellular uptake of Cy5-Luc-mRNA NPs (mRNA concentration: 0.25 μg/mL). This experiment was repeated thrice independently with similar results.

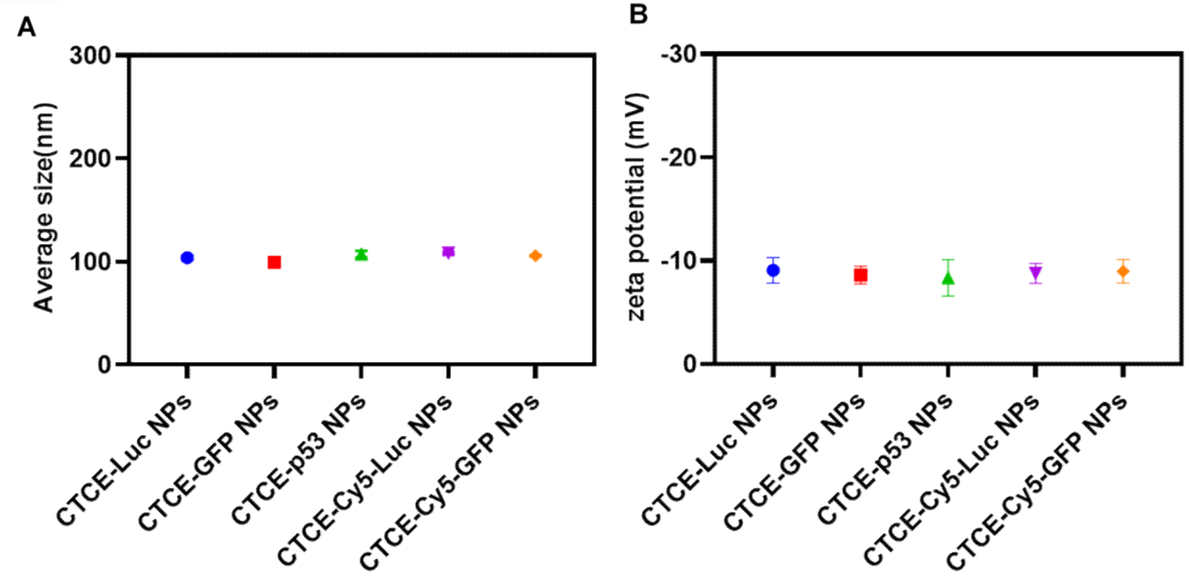

Supplementary Figure 6. (A) Average particle size (nm) and (B) zeta potential (mV) of the CTCE-Luc NPs, CTCE-GFP NPs CTCE-p53 NPs, CTCE-Cy5-Luc NPs and CTCE-Cy5-GFP NPs (n=3 samples/group). All data are presented as mean ± S.D.

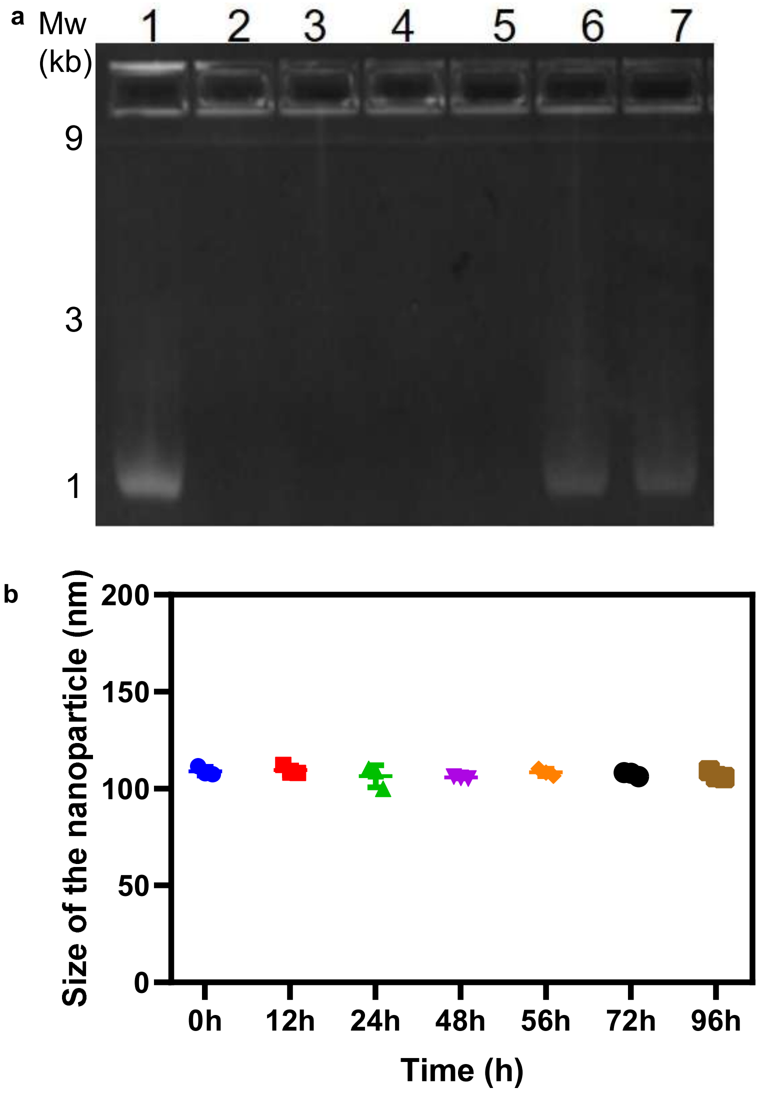

Supplementary Figure 7. Characterization of p53-mRNA NPs. **(a)** Agarose gel electrophoresis assay of EGFP-mRNA stability at various weight ratios of G0-C8/mRNA (Lane 1: 1; Lane 2: 2; Lane 3: 5; Lane 4: 10; Lane 5: 20) and at naked forms (Lane 6: free mRNA in DMF, Lane 7: free mRNA in 1X PBS buffer). This experiment was repeated thrice independently with similar results. **(b)** Stability of p53-mRNA NP in 10% serum at 37°C was evaluated by measuring particle size changes with dynamic light scattering at various time points up to 96h. This experiment was repeated thrice independently with similar results.

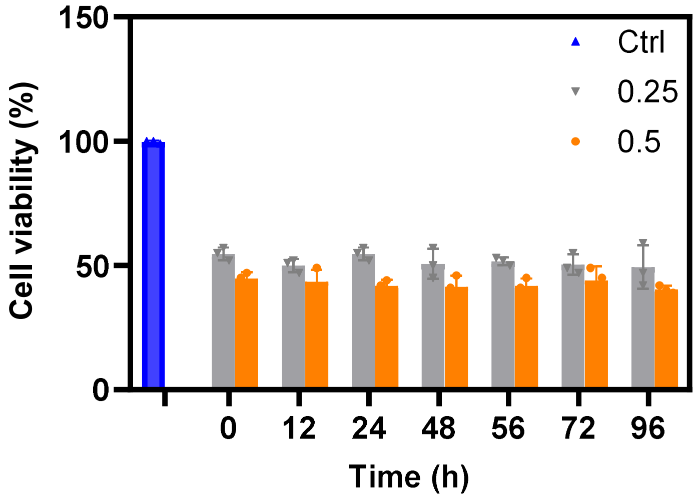

Supplementary Figure 8. Stability of p53-mRNA NP in 10% serum at 37°C evaluated by measuring cell viabilities towards RIL-175 cells at various time points up to 96h ((n=3 cell samples/group). All data are presented as mean ± S.D.

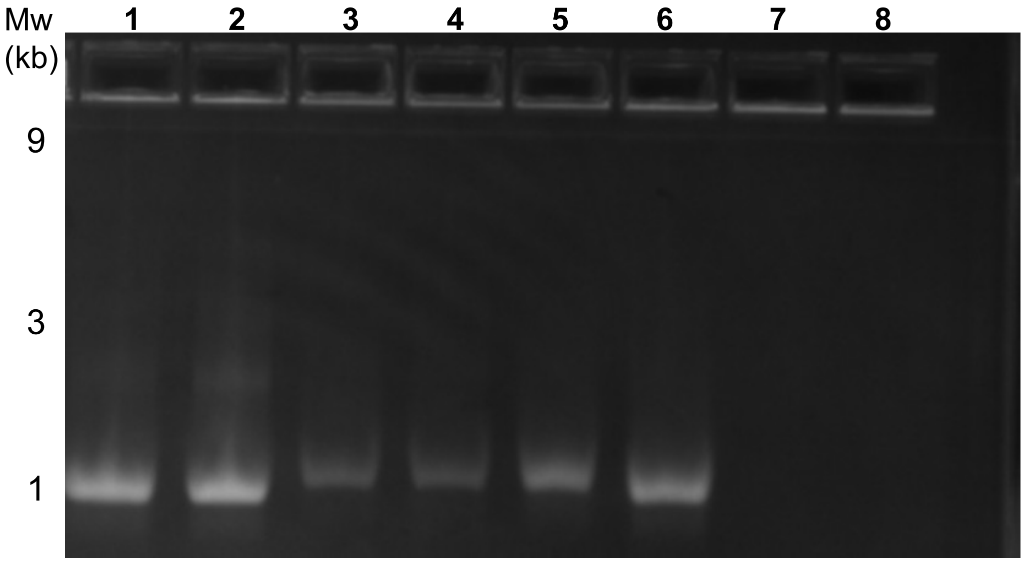

Supplementary Figure 9. EGFP-mRNA complexation ability of G0-C8 at different pH and weight ratios of G0-C8/mRNA by agarose gel electrophoresis assay (Lane 1: pH 7.4, G0-C8/mRNA= 2; Lane 2: pH 7.4, G0-C8/mRNA= 5; Lane 3: pH 7.4, G0-C8/mRNA= 50; Lane 4: pH 7.4, G0-C8/mRNA= 200; Lane 5: free mRNA in pH 7.4; Lane 6: free mRNA in pH 3.5; Lane 7: pH 3.5, G0-C8/mRNA= 2; Lane 8: pH 3.5, G0-C8/mRNA= 5). This experiment was repeated thrice independently with similar results.

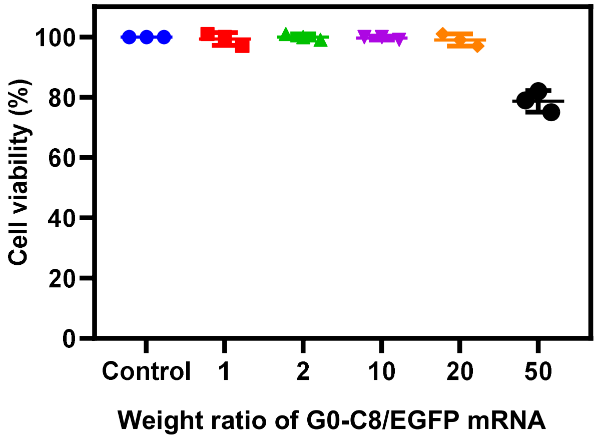

Supplementary Figure 10. *In vitro* cell viability of G0-C8 at different ratios of G0-C8/EGFP mRNA. All data are presented as mean ± S.D (n=3 cell samples/group).

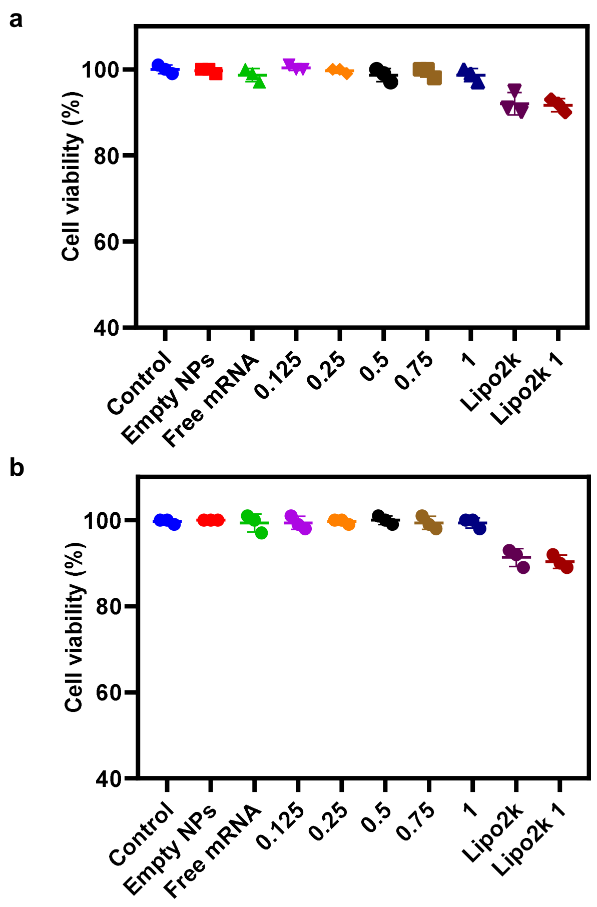

Supplementary Figure 11. *In vitro* toxicity study of Luciferase-mRNA NPs. The viability of the **(a)** p53-null RIL-175 cells and **(b)** normal liver cells THLE-3 cells after the treatment with PBS, empty NPs, free Luciferase-mRNA (1 μg/ml), CTCE-Luciferase-mRNA NPs (0.125, 0.25, 0.5, 0.75 or 1 μg/mL), Lipo2k (Lipo2k only) and Luciferase-mRNA Lipo2k 1 (Luciferase mRNA transfected by Lipo2k at the mRNA concentration of 1 μg/mL), as measured by AlamarBlue assay. All data are presented as mean ± S.D (n=3 cell samples/group).

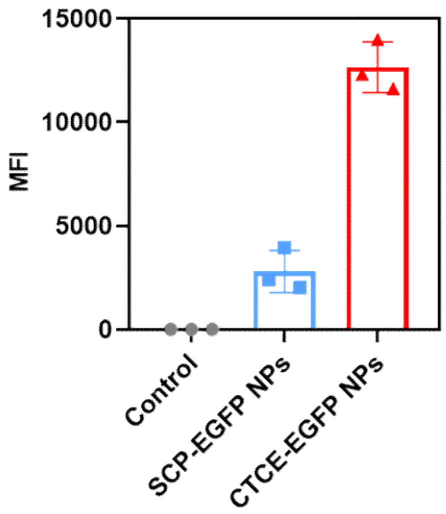

Supplementary Figure 12. Flow cytometry analysis of *in vitro* transfection efficiency (%GFP positive cells) of free EGFP mRNA, SCP-EGFP NPs and CTCE-EGFP NPs in *p53*-null RIL-175 cells: mean fluorescent intensity (MFI) in transfected cells analyzed by Flowjo. All data are presented as mean ± S.D (n=3 cell samples/group).

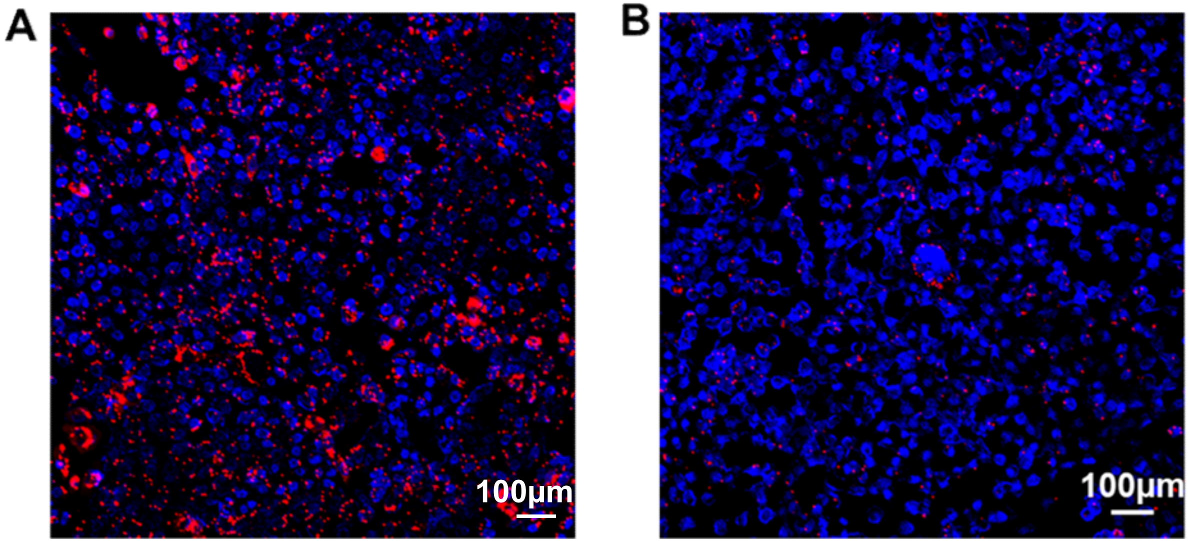

Supplementary Figure 13. (A) Fluorescent images of RIL-175 cells treated with CTCE-Cy5-Luciferase mRNA NPs. (B) Fluorescent images of RIL-175 cells after blocking the CXCR-4 receptor. This experiment was repeated thrice independently with similar results.

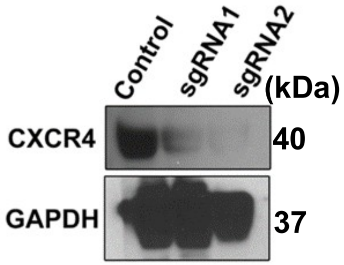

Supplementary Figure 14. Western blotting of the CXCR4 expression of the CXCR4-KO RIL-175 cells (sgRNA2) by CRISPR/Cas9 editing. This experiment was repeated thrice independently with similar results.

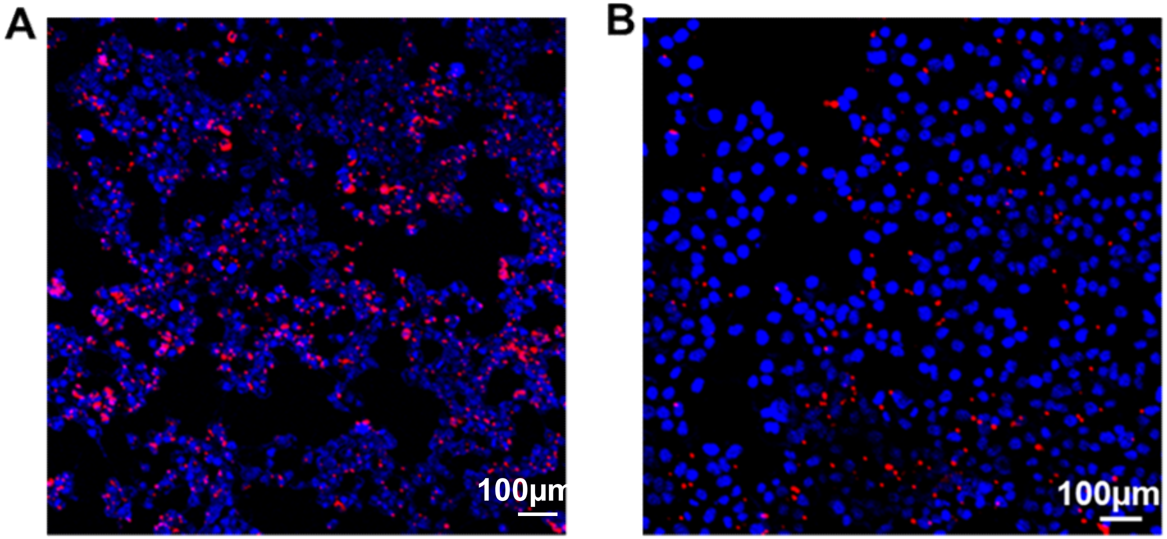

Supplementary Figure 15. (A) Fluorescent images of sg control RIL-175 cells treated with CTCE-Cy5-Luciferase mRNA NPs. (Right) Fluorescent images of CXCR4-KO cell line (sgRNA2 RIL-175 cells). This experiment was repeated thrice independently with similar results.

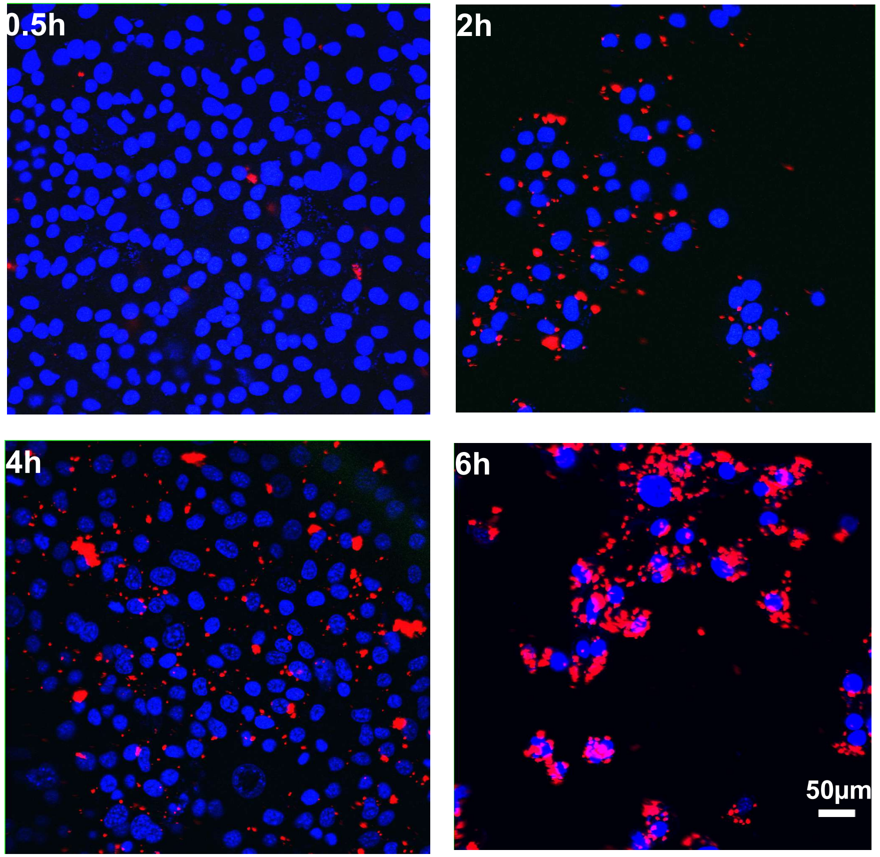

Supplementary Figure 16. Representative immunofluorescence of CTCE-Cy5-Luciferase mRNA NP cellular uptake at different time points. This experiment was repeated thrice independently with similar results.

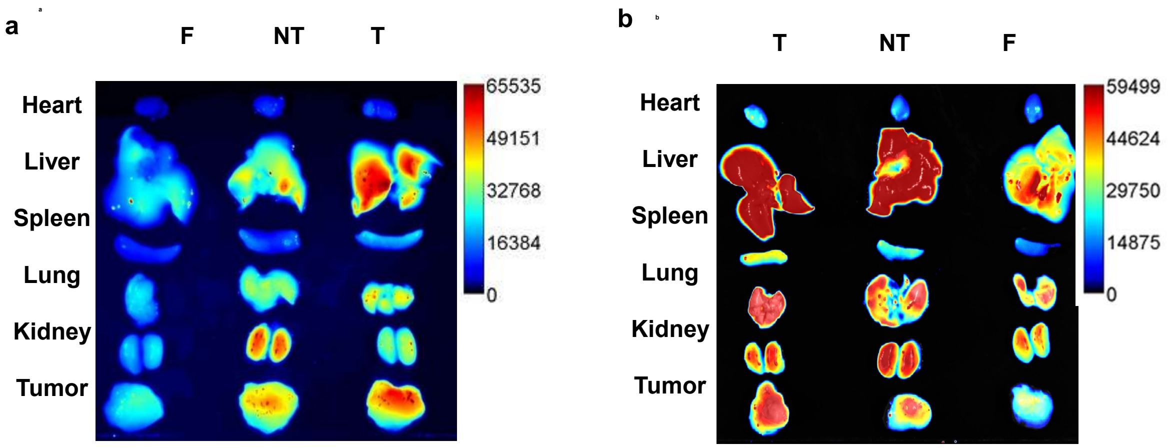

Supplementary Figure 17. Representative biodistribution of free Cy5-Luciferase mRNA (F), non-targeted Cy5-Luciferase mRNA NP (NT), and CTCE targeted Cy5-Luciferase-mRNA NP (T) in different organs including tumors from C57BL/6 mice bearing RIL-175 tumor 24h post-injection (mRNA dosage: 350 µg/kg). **(a)** Orthotopic RIL-175 HCC model, **(b)** s.c. grafted RIL-175 HCC model. Representative image from one of three independent experiments.

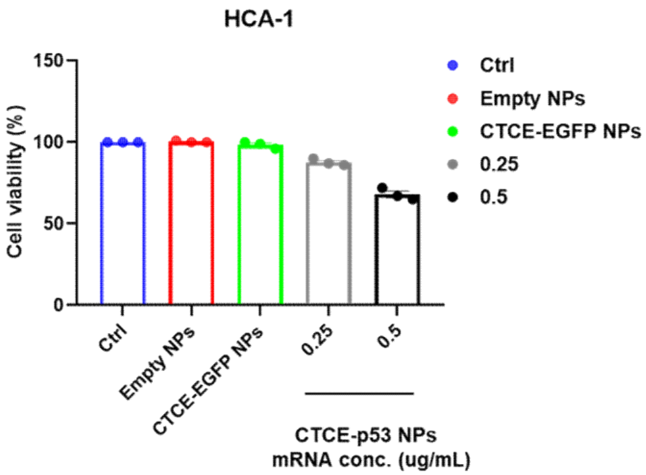

Supplementary Figure 18. HCA-1 cell viability after treatment with control (saline), Empty NPs, Control NPs (CTCE-EGFP NPs), or CTCE-p53 NPs with different mRNA concentrations (0.25 and 0.5 μg/mL, respectively). All data are presented as mean ± S.D (n=3 cell samples/group).

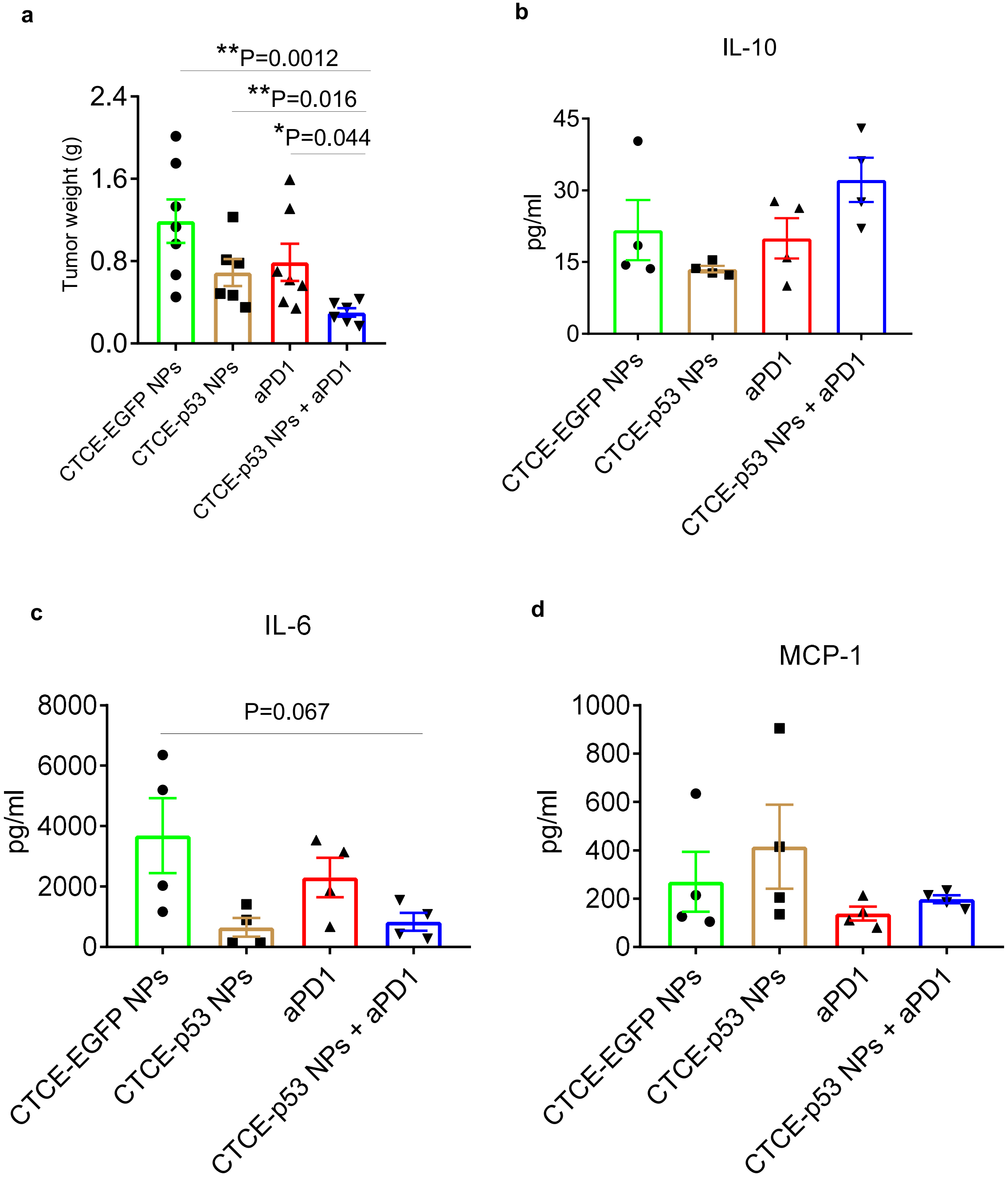

Supplementary Figure 19. PD1 blockade combined with CXCR4-targeted p53 mRNA NPs inhibits tumor growth and reprograms the immune TME. (a) Tumor weight after 4 injections of CTCE-EGFP mRNA NPs (i.v.) and/or anti-PD1 (aPD1) antibody (10mg/kg, i.p.) every 3 days (n=7 mice for CTCE-EGFP-NPs and aPD1 group; n=6 mice for CTCE-p53 NPs and CTCE-p53 NPs+ aPD1 group). (b-d) Multiplexed array analysis of tumor tissue for expression levels of IL-10 (b), IL-6 (c) and MCP1 (CCL2) (d) (n=4 tumor samples for each group). $^{*}p < 0.05$ from one-way ANOVA with a Tukey post-hoc test. All data are presented as mean ± S.E.M.

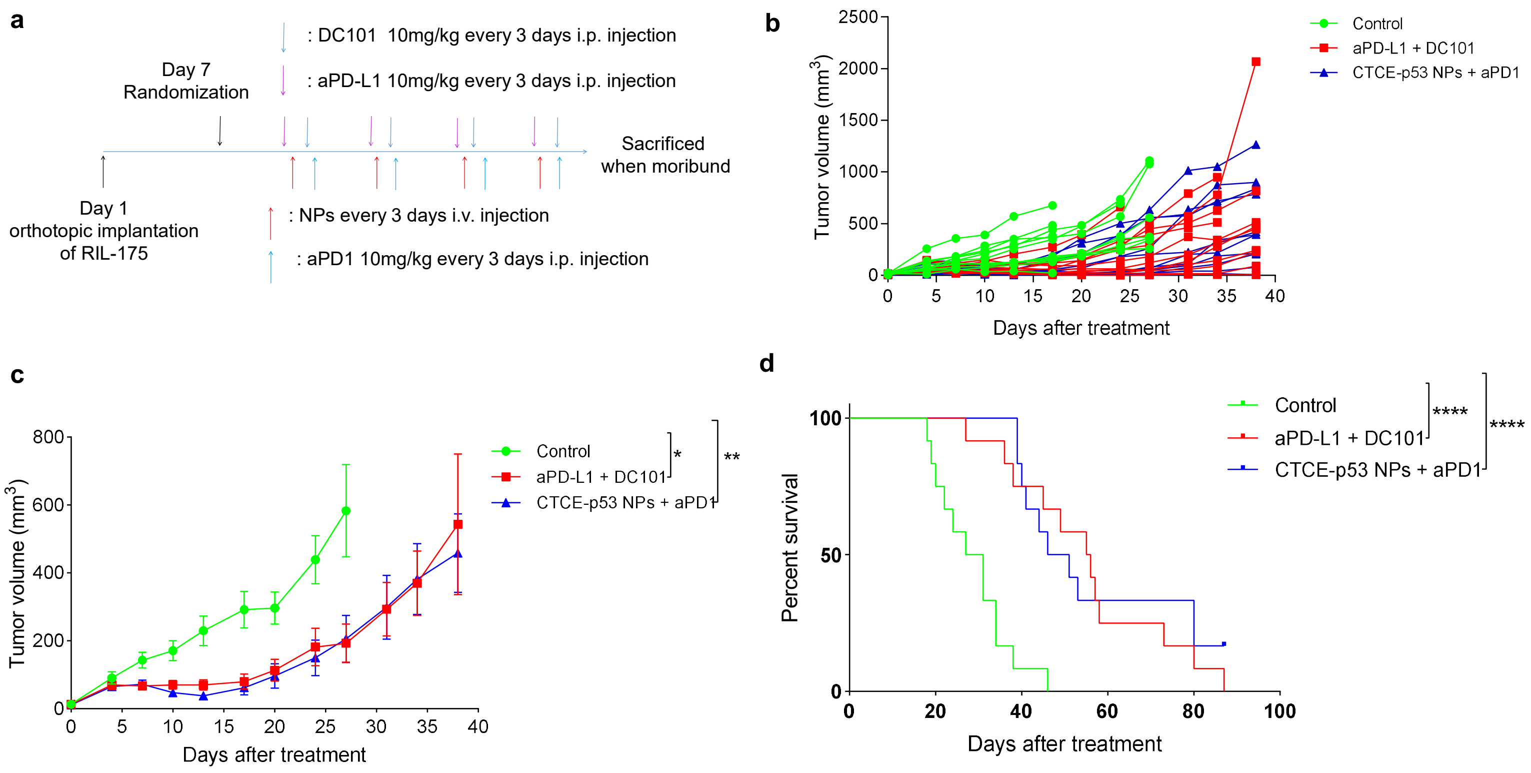

Supplementary Figure 20. The comparison of the therapeutic efficacy between the combination of CTCE-p53-mRNA NPs with anti-PD-1 (aPD1) and the combined treatment of anti-PD-L1 (aPD-L1) and anti-VEGFR2 (DC101) in orthotopic HCC model in C57BL/6 mice. **(a)** Timeline of tumor implantation and treatment schedule for survival studies in RIL-175 orthotopic murine HCC model. To develop the RIL-175 orthotopic model, approximately 6×10^5 RIL-175 cells 1:1 in Matrigel (Mediatech/Corning, Manassas, VA) were grafted into the left extrahepatic lobe of C57Bl/6 mice (6-8 weeks old). .**(b, c)** Tumor growth kinetics in each treatment group measured by ultrasound imaging, data are presented as mean ± S.E.M. **(d)** Survival distributions in each treatment group. For Figures b-d, day 0: the time for the first treatment. VEGFR2: Vascular Endothelial Growth Factor Receptor 2. Statistical significance was analyzed via one-way ANOVA with a Tukey post-hoc test. The statistical method for survival analysis is Logrank test. *P<0.05; **P<0.01; ****P<0.0001; n=12 mice/group.

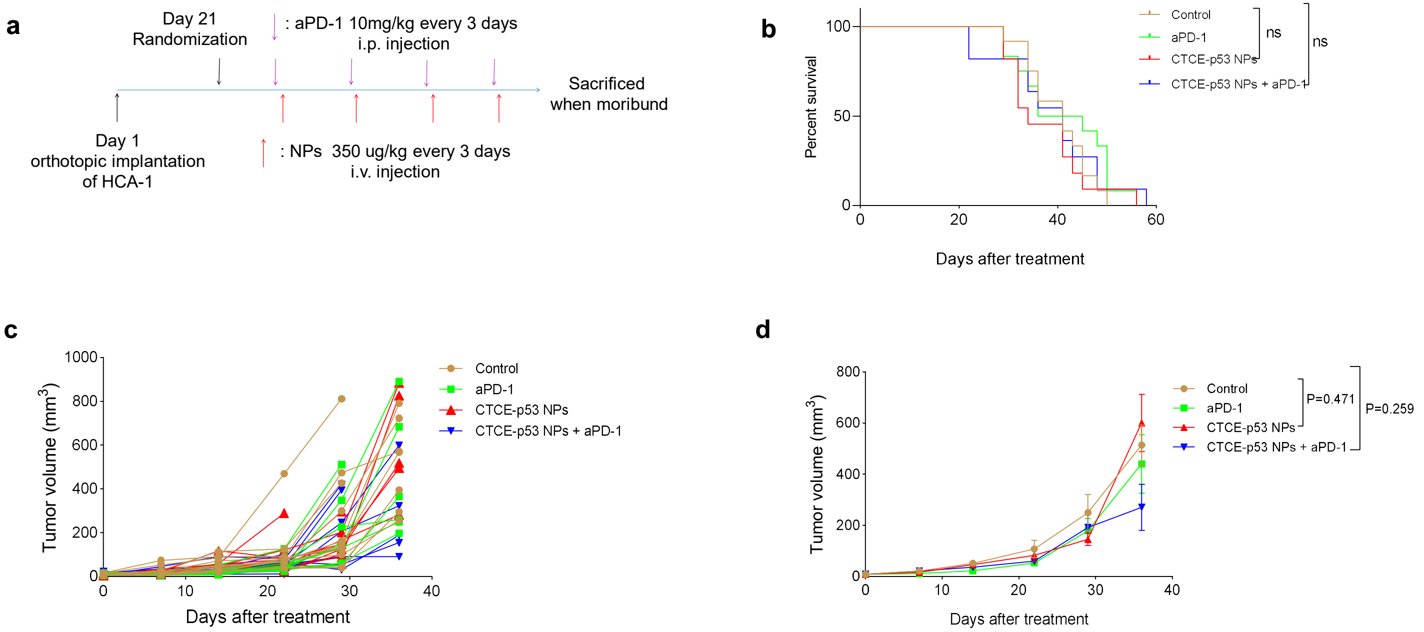

Supplementary Figure 21. The therapeutic efficacy of the combination of CTCE-p53-mRNA NPs with anti-PD-1 (aPD1) in C3H mice via orthotopic HCA-1 HCC model. **(a)** Timeline of tumor implantation and treatment schedule for survival studies in HCC models. **(b)** Survival data from the HCA-1 orthotopic mice model (n=12). **(c, d)** Tumor growth profile of each indicated treatment group (n=12 mice for CTCE-EGFP-NPs and aPD1 group; n=11 mice for CTCE-p53 NPs and CTCE-p53 NPs+ aPD1 group). Statistical significance was analyzed via one-way ANOVA with a Tukey post-hoc test. Data are presented as mean ± S.E.M. *P<0.05; **P<0.01; ****P<0.0001.

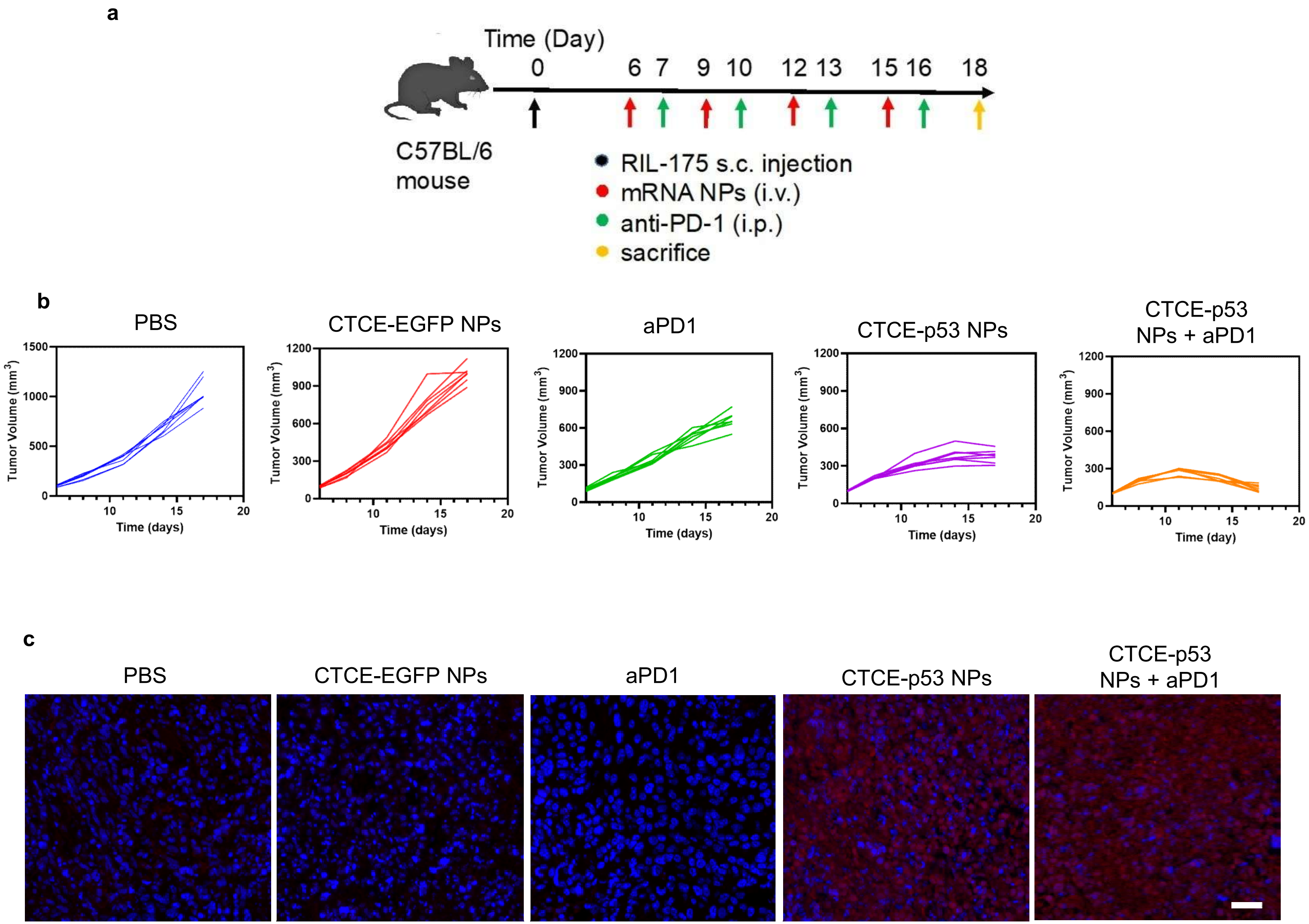

Supplementary Figure 22. Anti-tumor efficacy of p53-mRNA NPs with anti-PD-1 in a p53-null ectopically (subcutaneously) grafted RIL-175 HCC model. (a) Experimental design and treatment schedule (n=12 mice/group). (b) Individual tumor growth curves for mice treated with indicated formulations. (n=7 mice/group). (c) Representative immunofluorescence for p53 protein expression in tumor tissues after different treatments. Scale bar: 200μm. This experiment was repeated thrice independently with similar results.

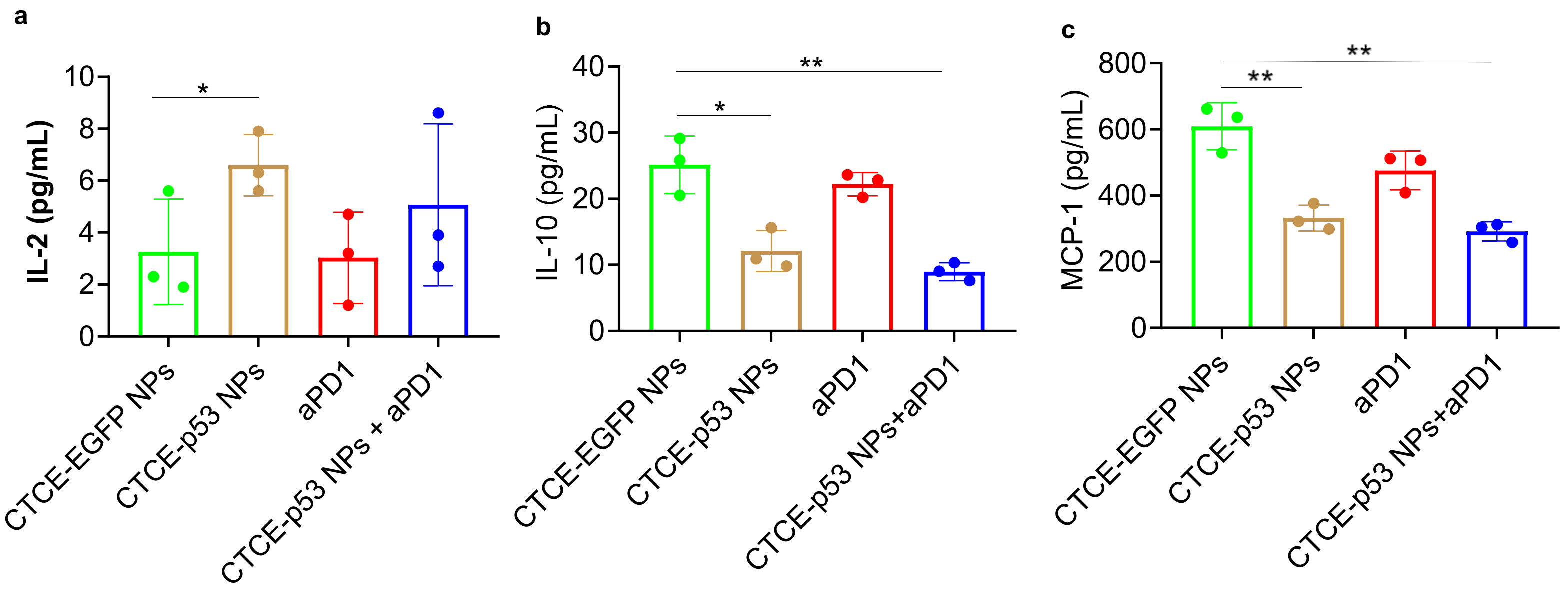

Supplementary Figure 23. Protein array analysis of cytokines tumor tissues after treatment in the s.c. grafted HCC model (n=3 tumor samples for each group): (a) IL-2, (b) IL-10, (c) MCP-1. Statistical significance was calculated via one-way ANOVA with a Tukey post-hoc test. All data are presented as mean ± S.E.M. *P<0.05; **P<0.01; ***P<0.001.

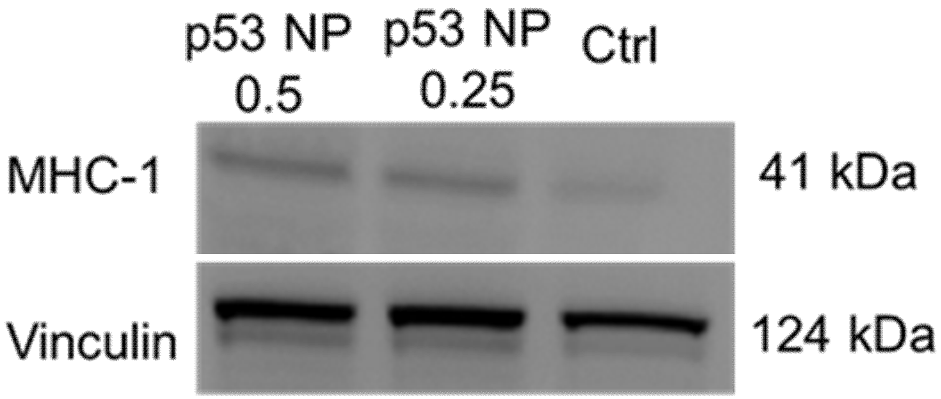

Supplementary Figure 24. Western blot analysis of MHC-1 expression in RIL-175 tumor cell line after p53 NPs treatment at p53 mRNA concentration of 0.25 μg/mL (p53 NP 0.25) and 0.5 μg/mL (p53 NP 0.5), respectively. This experiment was repeated thrice independently with similar results.

.

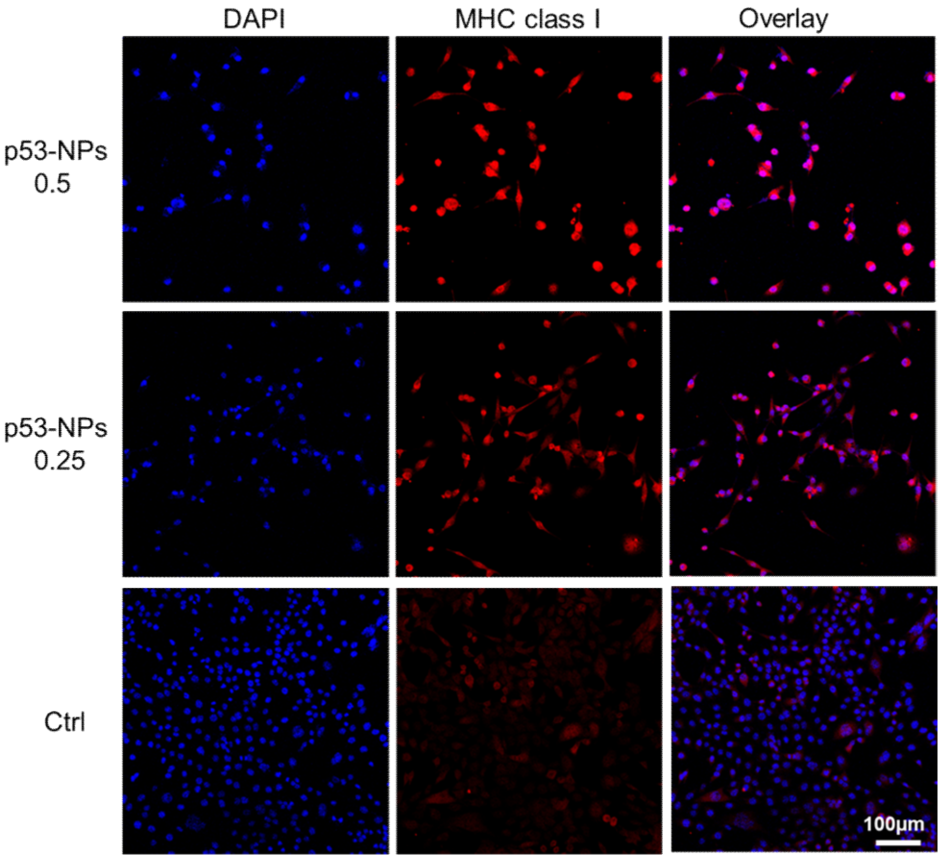

Supplementary Figure 25. Immunofluorescence microscopic images of MHC class 1 expression in RIL-175 tumor cell line after p53 mRNA NPs treatment at p53 mRNA concentration of 0.25 μg/mL and 0.5 μg/mL, respectively. This experiment was repeated thrice independently with similar results.

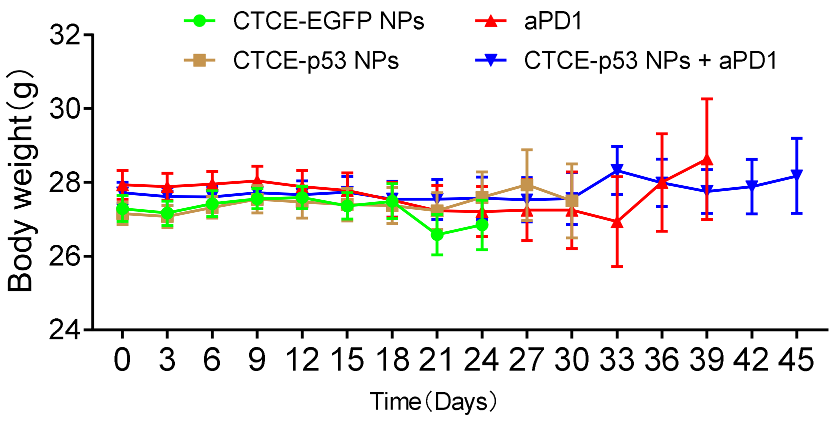

Supplementary Figure 26. Average body weight of RIL-175 orthotopic tumor-bearing mice over the course of therapy. Error bars represent body weight in grams ± S.E.M. (n=12 mice /group).

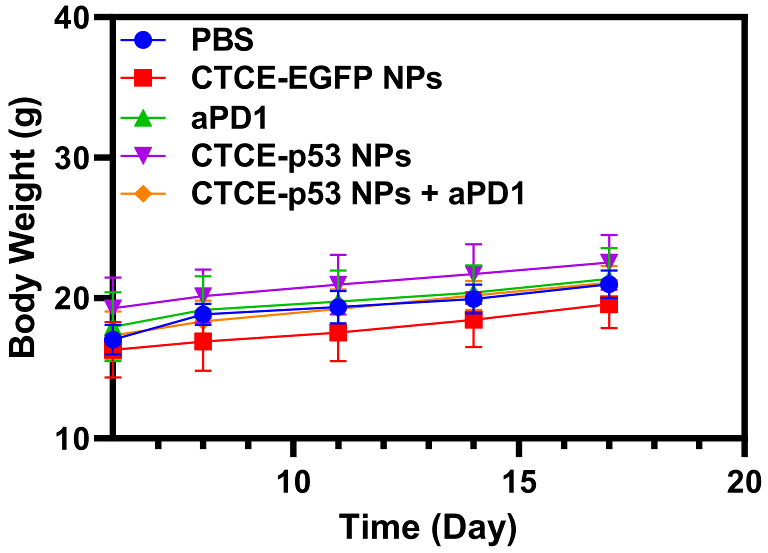

Supplementary Figure 27. Average body weight of RIL-175 s.c. tumor-bearing mice over the course of therapy. Error bars represent the S.E.M. (n=7 mice/group).

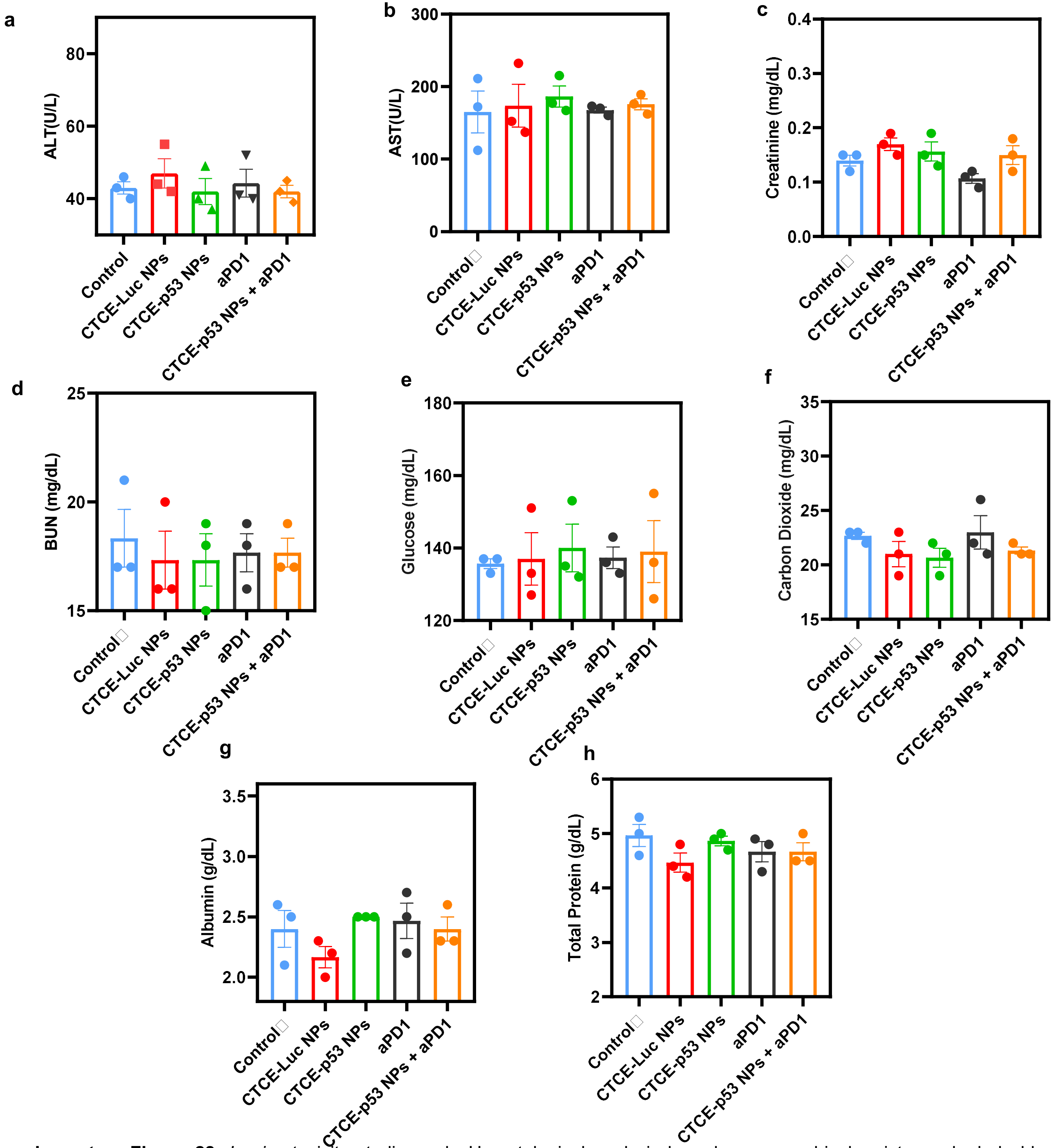

Supplementary Figure 28. *In vivo* toxicity studies. a-h, Hematological analysis based on serum biochemistry and whole blood panel tests. The following parameters were examined: alanine aminotransferase (ALT) (a), aspartate aminotransferase (AST) (b), creatinine (c), urea nitrogen (BUN) (d), glucose (e), carbon dioxide (f), albumin (g), total protein (h) in HCC tissues in the s.c. RIL-175 model. (n=3 mice/group). All data are presented as mean ± S.E.M.

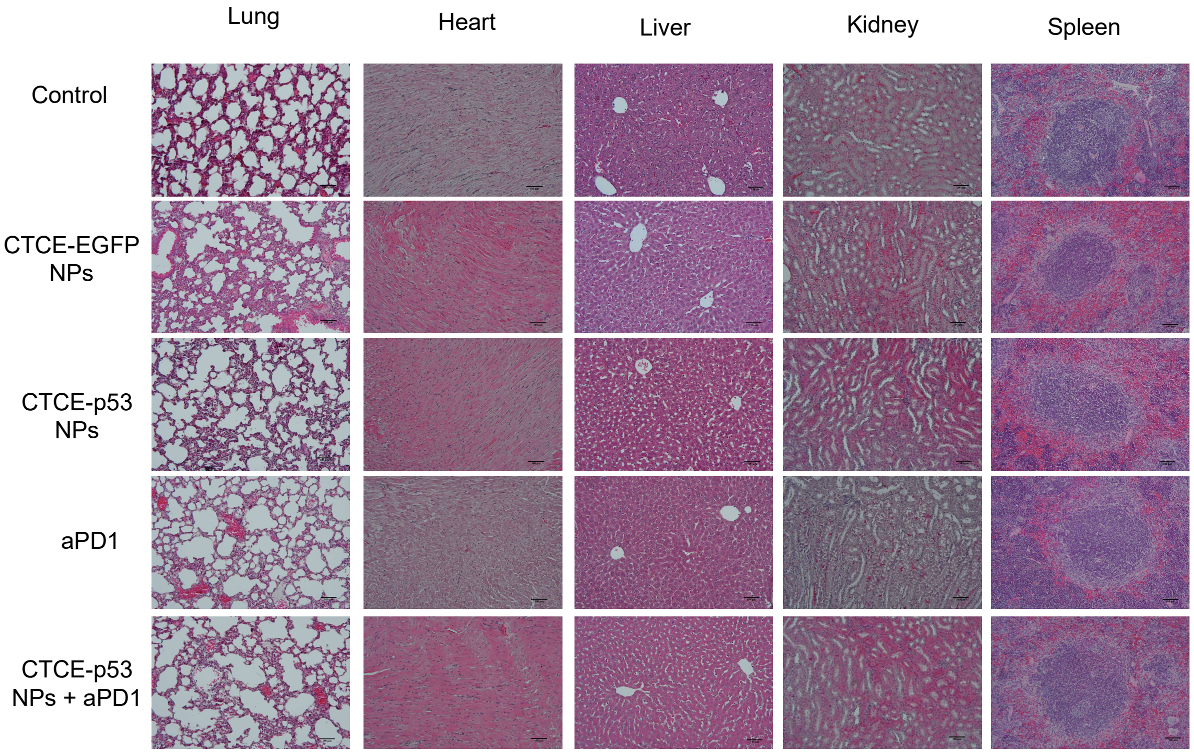

Supplementary Figure 29. Representative H&E staining of heart, liver, spleen, kidney, and lung tissues after treatment with PBS, CTCE-EGFP NPs, aPD1, CTCE-p53 NPs and CTCE-p53 NPs combined with aPD1 in orthotopic RIL-175 tumor model. Bar: 100μm. This experiment was repeated thrice independently with similar results.

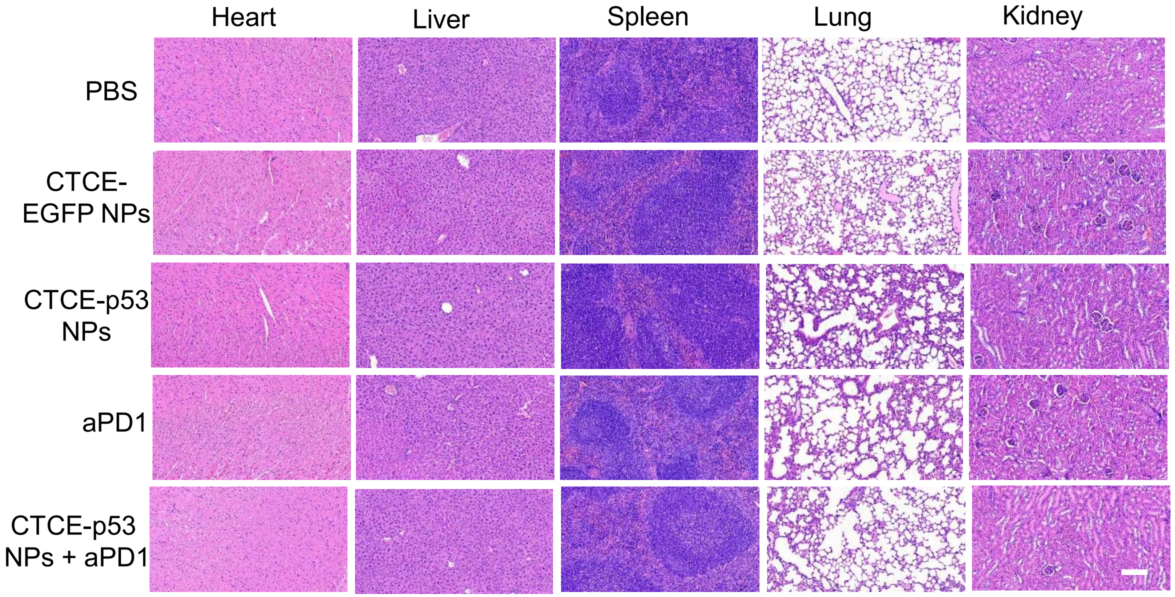

Supplementary Figure 30. Representative H&E staining of heart, liver, spleen, kidney, and lung tissues after treatment with PBS, CTCE-EGFP NPs, aPD1, CTCE-p53 NPs and CTCE-p53 NPs combined with aPD1 in s.c. RIL-175 tumor model. Scale bar: 200μm. This experiment was repeated thrice independently with similar results.

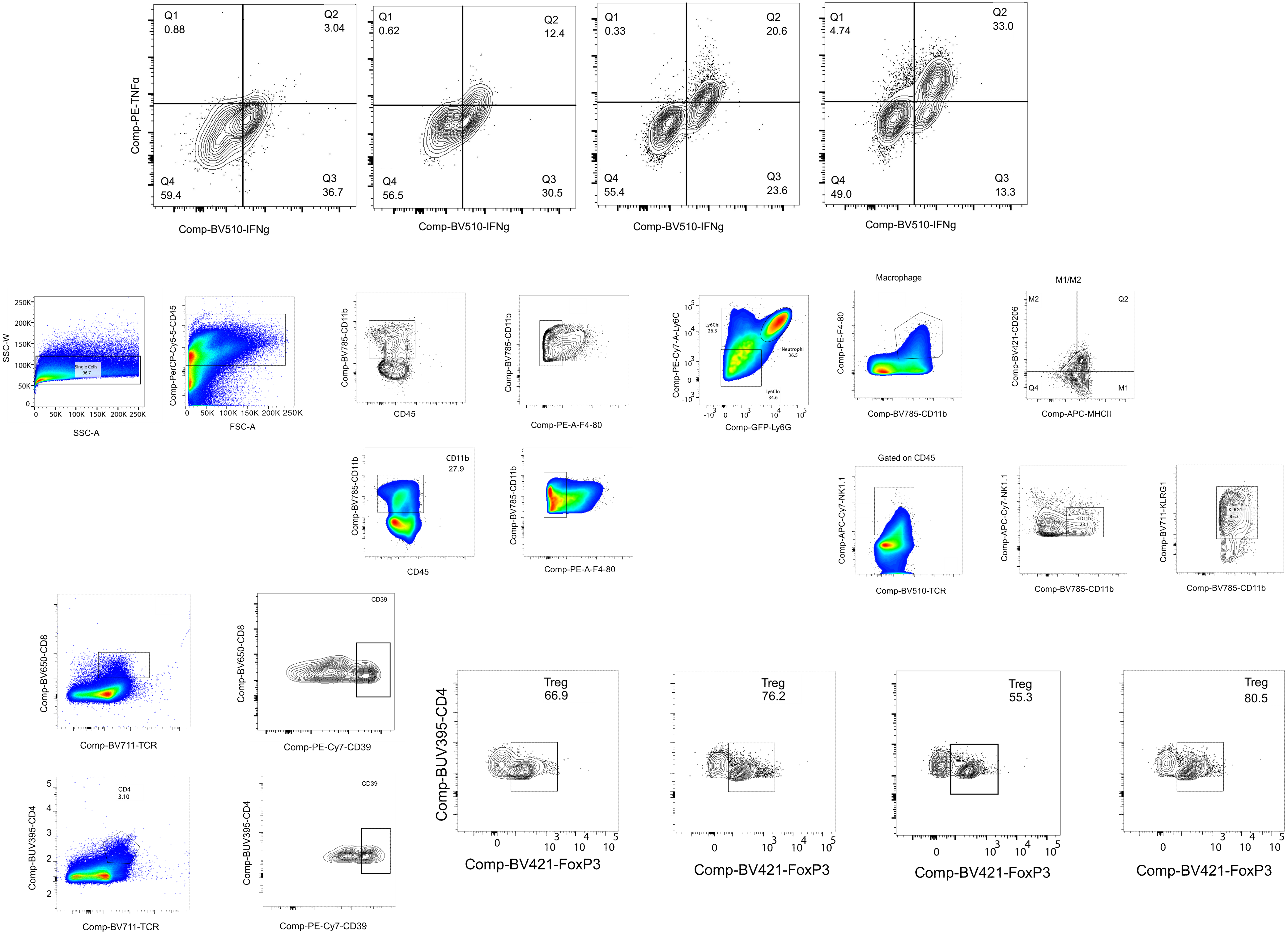

Supplementary Figure 31. Flow Cytometry Gating strategy.

Supplementary Table 1. Effect of different cationic lipid-like materials G0-Cm on the encapsulation efficacy of Cy5-Luciferase mRNA NPs.

	G_0-C_8	G_0-C_{10}	G_0-C_{12}	G_0-C_{14}	G_0-C_{16}
**Encapsulation efficiency(EE) %**	67.3	65.9	63.7	66.9	58.3
