# Supplementary material for: Combining p53 mRNA nanotherapy with immune checkpoint blockade reprograms the immune microenvironment for effective cancer therapy
Source: Nat Commun. 2022 Feb 9;13:758. doi: 10.1038/s41467-022-28279-8 (PMC8828745; doi:10.1038/s41467-022-28279-8)
Supplement: Supplementary file 2 — Reporting Summary [file 41467_2022_28279_MOESM2_ESM.pdf]

nature research

Corresponding author(s): Dan G. Duda, Jinjun Shi

Last updated by author(s): Dec 26, 2021

Reporting Summary

Nature Research wishes to improve the reproducibility of the work that we publish. This form provides structure for consistency and transparency in reporting. For further information on Nature Research policies, see our Editorial Policies and the Editorial Policy Checklist.

Statistics

For all statistical analyses, confirm that the following items are present in the figure legend, table legend, main text, or Methods section.

n/a	Confirmed	
☐	☒	The exact sample size (n) for each experimental group/condition, given as a discrete number and unit of measurement
☐	☒	A statement on whether measurements were taken from distinct samples or whether the same sample was measured repeatedly
☐	☒	The statistical test(s) used AND whether they are one- or two-sided<br>*Only common tests should be described solely by name; describe more complex techniques in the Methods section.*
☒	☐	A description of all covariates tested
☒	☐	A description of any assumptions or corrections, such as tests of normality and adjustment for multiple comparisons
☐	☒	A full description of the statistical parameters including central tendency (e.g. means) or other basic estimates (e.g. regression coefficient) AND variation (e.g. standard deviation) or associated estimates of uncertainty (e.g. confidence intervals)
☐	☒	For null hypothesis testing, the test statistic (e.g. F, t, r) with confidence intervals, effect sizes, degrees of freedom and P value noted<br>*Give P values as exact values whenever suitable.*
☒	☐	For Bayesian analysis, information on the choice of priors and Markov chain Monte Carlo settings
☒	☐	For hierarchical and complex designs, identification of the appropriate level for tests and full reporting of outcomes
☒	☐	Estimates of effect sizes (e.g. Cohen's d, Pearson's r), indicating how they were calculated

Our web collection on statistics for biologists contains articles on many of the points above.

Software and code

Policy information about availability of computer code

Data collection	Microsoft Excel: version 2016; NMR spectra: Samples were acquired on Varian MR 400MHz Automated NMR System<br>Cell viability assay/ Colorimetric assay(absorbance): Microplate reader (TECAN, Infinite M200 Pro)<br>In vitro EGFP mRNA transfection: Fluorescence microscopy (Axiovert 200, Zeiss)<br>Cellular uptake: Confocal laser scanning microscopy (Olympus, FV1100)<br>FACS analysis: Samples were acquired by DXP11 Analyzer (BD Biosystems), Accuri C6 Plus (BD Biosciences) and FACS Aria cell so .<br>rter Western blot: immunostained bands were detected under the Syngene PXi imaging system (Synoptics Ltd)<br>In vivo bioluminescent imaging: Bruker Xtreme scanner (Bruker), Bruker MI 7.5.2<br>High-frequency ultrasonography: VisualSonics Vevo2100 (FUJI FILM).
Data analysis	Chemical structures were drawn by Chemdraw 14.<br>The NMR s pectra were analyzed by Mnova 14.2.0.<br>For FACS analysis data were processed by Flowjo software (Flowjo V10).<br>For western blot bands intensity was quantified with NIH Image J software (version 1.52a).<br>For in vivo bioluminescent imaging data were analyzed by Bruker MI SE.<br>For statistical analysis and graphic representation: GraphPad Prism v8.0.1.

For manuscripts utilizing custom algorithms or software that are central to the research but not yet described in published literature, software must be made available to editors and reviewers. We strongly encourage code deposition in a community repository (e.g. GitHub). See the Nature Research guidelines for submitting code & software for further information.

Data

Policy information about availability of data

All manuscripts must include a data availability statement. This statement should provide the following information, where applicable:

- Accession codes, unique identifiers, or web links for publicly available datasets
- A list of figures that have associated raw data
- A description of any restrictions on data availability

All relevant data are included in the paper and/ or its supplementary information files.

Field-specific reporting

Please select the one below that is the best fit for your research. If you are not sure, read the appropriate sections before making your selection.

[x] Life sciences [ ] Behavioural & social sciences [ ] Ecological, evolutionary & environmental sciences

For a reference copy of the document with all sections, see nature.com/documents/nr-reporting-summary-flat.pdf

Life sciences study design

All studies must disclose on these points even when the disclosure is negative.

Sample size	No sample size calculations were performed. Animal sample size was determined by the number of biological replicates necessary for ensuring statistical significance. The number of biological replicates are also reported in the relevant figure legends in the manuscript. For experiments other than animal studies, the sample size (n) of each experiment is provided in the figure captions in the manuscript and supplementary information accordingly. Sample sizes were chosen to support meaningful conclusions. In addition, we adhere to sample size requirements necessary for determining statistical significance.
Data exclusions	No data were excluded.
Replication	All attempts at replication were successful. Experimental repeat numbers are also reported in Figure Legends.
Randomization	All samples were randomly allocated into experimental groups.
Blinding	Experiments were generally blinded.

Reporting for specific materials, systems and methods

We require information from authors about some types of materials, experimental systems and methods used in many studies. Here, indicate whether each material, system or method listed is relevant to your study. If you are not sure if a list item applies to your research, read the appropriate section before selecting a response.

Materials & experimental systems

n/a	Involved in the study	
[ ]	[x]	Antibodies
[ ]	[x]	Eukaryotic cell lines
[x]	[ ]	Palaeontology and archaeology
[ ]	[x]	Animals and other organisms
[x]	[ ]	Human research participants
[x]	[ ]	Clinical data
[x]	[ ]	Dual use research of concern

Methods

n/a	Involved in the study	
[x]	[ ]	ChIP-seq
[ ]	[x]	Flow cytometry
[x]	[ ]	MRI-based neuroimaging

Antibodies

Antibodies used	All the antibodies (name, vendor, catalog, dilution factors) were provided in the method section (under Materials and Flow cytometry and cytokine analysis Sections). Anti-p53 (sc-126, Santa Cruz Biotechnology, 1:500 dilution), Anti-GAPDH (Cell Signaling Technology, 5174; 1:2,000 dilution), anti-beta-Actin (Cell Signaling Technology; 4970, 1: 2,000 dilution), anti-rabbit and anti-mouse horseradish peroxidase (HRP)-conjugated secondary antibodies were obtained from Cell Signaling Technology (7074, 7076). Secondary antibodies used in this study included: Alexa Fluor® 488 Goat-anti Rabbit IgG (Life Technologies, A-11034), and Alexa Fluor® 647 Goat-anti Mouse IgG (Life Technologies, A-28181). Fluorescence-labelled antibodies CD11c (Biolegend, cat. no. 117310, clone N418), CD80 (Biolegend, cat. no. 104722, clone 16-10A1), CD 86 (Biolegend, cat. no. 105005, clone clone GL-1), CD4 (Biolegend, cat. no. 100412, clone GK1.5), CD3 (Biolegend, cat. no. 100204, clone 17A2), CD8 (Biolegend, cat. no. 140408, clone 53–5.8), CD8 (Biolegend, cat. no. 100741, clone 53–6.7), CD11b (Biolegend, cat. no. 101208, clone M1/70), F4/80 (Biolegend, cat. no. 123116, clone BM8), CD206 (Biolegend, cat. no. 141716, clone C068C2), Gr-1 (Biolegend, cat. no. 108412, clone RB6-8C5), TNFa (Biolegend, cat. no. 506305, clone MP6-XT22), Ly6C

(Biolegend, cat. no. 128005, clone HK1.4), CD45 (Biolegend, cat. no. 103108, clone 30-F11), NK1.1 (Biolegend, cat. no. 156509, clone S17016D), TCR (Biolegend, cat. no. 109243, clone H57-597), KLRG1 (Biolegend, cat. no. 138427, clone 2F1/KLRG1), CD39 (Biolegend, cat. no. 143805, clone Duha59), Ki67 (Biolegend, cat. no. 652423, clone 16A8), GzmB (Biolegend, cat. no. 372203, clone QA16A02), CD11b (Biolegend, cat. no. 101243, clone M1/70), CD206 (Biolegend, cat. no. 141717, clone C068C2), Forkhead box protein P3 (FoxP3; Biolegend, cat. no. 126419, clone MF-14), IFN-γ Receptor βchain (Biolegend, cat. no. 113605, clone MOB-47), IFNG (Biolegend, cat. no. 505841, clone XMG1.2), CD119 (BD Bioscience, cat. no. 740897, clone GR20), FITC (Biolegend, cat. no. 503805, clone JES6-5H4) following the manufacturer's instructions. All these antibodies were diluted 200 times.

Validation

All primary antibodies were bought from vendors (BD, biolegend, ebioscience, abcam and cell signaling technology) and used for the specifies suggested by the manufacturers. All the antibodies were validated by the suppliers. Validations can be found in the manufacturer's website. In addition, many have literature references and species specifications on their websites. CD4, CD8, CD11c, CD80, CD86 et al. are routinely used antibodies. Dilution factors were pre-determined in the lab. Flow data suggests clear isolation of the cell populations.

Eukaryotic cell lines

Policy information about cell lines

Cell line source(s)

Cell line sources were provided under "Method: Cell culture" section. The luciferase-expressing p53-null murine HCC cell line RIL-175 was used throughout the study. RIL-175 (a p53-null/Hras mutant line syngeneic to C57Bl/6 mouse strain background, Luciferase-tagged) was kindly provided by Dr. Tim Greten (NIH). HEK 293T cells were purchased from American Type Culture Collection (ATCC).

Authentication

The cell lines were certified by the manufacturers (surface markers, morphology).

Mycoplasma contamination

Cell lines were all tested negative for mycoplasma contamination llonza MycoAiert kit.

Commonly misidentified lines
(See ICLAC register)

No commonly misidentified cell lines were used.

Animals and other organisms

Policy information about studies involving animals; ARRIVE guidelines recommended for reporting animal research

Laboratory animals

Laboratory animals were provided under "Method: Animals" section. Immunocompetent male and female C57BL/6 mice (5-6 weeks old or 6-8 weeks old) were obtained from the Charles River Laboratories and housed in a pathogen-free animal facility of Brigham and Women's Hospital or Massachusetts General Hospital. All animals were housed in single-unit cages with 12-hr alternate light and dark cycles and at controlled ambient temperature (68-79 F) with humidity between 30%-70%.

Wild animals

No wild animals were used in this study.

Field-collected samples

The samples did not involve samples collected from the field.

Ethics oversight

For orthotopic tumor model, all animal experiments were performed after approval by the Institutional Animal Care and Use Committee of the Massachusetts General Hospital. For s.c. tumor model, all animal procedures were performed with ethical compliance and approval by the Institutional Animal Care and Use Committees at Harvard Medical School.

Note that full information on the approval of the study protocol must also be provided in the manuscript.

Flow Cytometry

Plots

Confirm that:

- [x] The axis labels state the marker and fluorochrome used (e.g. CD4-FITC).
- [x] The axis scales are clearly visible. Include numbers along axes only for bottom left plot of group (a 'group' is an analysis of identical markers).
- [x] All plots are contour plots with outliers or pseudocolor plots.
- [x] A numerical value for number of cells or percentage (with statistics) is provided.

Methodology

Sample preparation

Tumor immune-environment response were accessed in the s.c. grafted and orthotopic HCC models in mice by flow cytometry after treatment. RIL-175 tumor bearing C57Bl/6 mice were systemically injected CTCE targeted p53 mRNA NPs or control groups every 3 days for four injections. Forty eight hours post treatment, the mice were euthanized, and the tumor tissue was harvested and then homogenized for flow cytometry. Tumor tissues were resected and minced, and fragments were incubated in HBSS with 1.5 mg/mL of hyaluronidase and 15 μg/mL of collagenase for 30 minutes at 37°C. Digested tissues were passed through a 70-μm cell strainer and washed twice with phosphate-buffered saline (PBS)/0.5% bovine serum albumin. Prior to immunostaining, cells were washed with the buffer and fixed and permeabilized with FoxP3/Transcription Factor Staining Buffer Set (eBioscience/Thermo Fischer Scientific) to stain the intracellular markers. Harvested cells were incubated in Dulbecco's Modified Eagle Medium with cell activation cocktail with brefeldin A (Biolegend) for 6 hr at 37°C. The cells were stained with the antibodies of cell surface and intracellular marker in the buffer with brefeldin A.

Instrument

The stained cells were measured on flow cytometers (Accuri C6 Plus, BD Biosciences and FACS Aria cell sorter)

Software	The stained cells were analyzed by FlowJo software (Flowjo V10).
Cell population abundance	The numbers presented in the flow cytometry analysis images are percentage based.
Gating strategy	Based on the pattern of FSC-A/SSC-A, cells were used for analysis of tumor immune microenvironment. Singlets were gated according to the pattern of FSC-H vs FSC-A. Positive populations were determined by the specific antibodies, which were distinct form negative populations. For s.c model, gating strategies are referred to those described in the BD website (https://www.bio-rad-antibodies.com/flow-cytometry-gating- strategies.html). Gating was first based on FSC/SSC together with viability dyes and singlet populations. The cell populations within the gate were further analysized based on expression of markers.

☒ Tick this box to confirm that a figure exemplifying the gating strategy is provided in the Supplementary Information.
